# Analysis of geometric morphometrics and molecular phylogeny for *Anopheles* species in the Republic of Korea

**DOI:** 10.1038/s41598-023-49536-w

**Published:** 2023-12-12

**Authors:** Jiseung Jeon, Heung Chul Kim, Terry A. Klein, Kwang Shik Choi

**Affiliations:** 1 School of Life Sciences, BK21 FOUR KNU Creative BioResearch Group, Kyungpook National University, Daegu, 41566 Republic of Korea; 2 School of Life Sciences, College of Natural Sciences, Kyungpook National University, Daegu, 41566 Republic of Korea; 3 Research Institute for Dok-do and Ulleung-do Island, Kyungpook National University, Daegu, 41566 Republic of Korea; 4 U Inc., Daesakwan-ro 34-gil, Yongsan-gu, Seoul, 04409 Republic of Korea; 5 Force Health Protection and Preventive Medicine, Medical Department Activity-Korea/65th Medical Brigade, Unit 15281, Pyeongtaek, APO AP 96281-5281, USA; 6 Present Address: PSC 450, Box 75R, Pyeongtaek, APO AP 96206, USA; 7 Research Institute for Phylogenomics and Evolution, Kyungpook National University, Daegu, 41566 Republic of Korea

**Keywords:** Evolution, Zoology

## Abstract

Human malaria, transmitted by *Anopheles* mosquitoes, is the most predominant mosquito-borne disease that is responsible for hundreds of thousands of deaths worldwide each year. In the Republic of Korea (ROK), there are currently several hundred malaria cases annually, mostly near the demilitarized zone (DMZ). Eight species of *Anopheles* mosquitoes are currently known to be present in the ROK. Similar to other major malaria vectors in Africa and India, it is very challenging to morphologically differentiate *Anopheles* mosquitoes in the ROK due to their extremely similar morphology. In this study, wing geometric morphometrics (WGM) were used to differentiate the eight *Anopheles* species collected at six locations near the DMZ, Seoul and Pyeongtaek from April–October 2021. Phylogenetic analysis was also performed using cytochrome *c* oxidase subunit 1 (*COI*), internal transcribed spacer 2 (ITS2), and tyrosine hydroxylase (*TH*) genes for comparison with WGM analysis and to infer evolutionary relationships. The results of cross-validation (overall accuracy = 74.8%) demonstrated that species identification using WGM alone was not possible with a high accuracy for all eight species. While phylogenetic analyses based on the *COI* region could not clearly distinguish some species, the analysis based on ITS2 and *TH* was more useful for resolving the phylogenetic correlation of the eight species. Our results may improve *Anopheles* species identification strategies for effective identification and control of malaria vectors in the ROK.

## Introduction

Malaria, a mosquito-borne disease transmitted by *Anopheles* spp. causes more than 600,000 deaths annually^1^. The primary mosquito vectors of the five human malarias belong to the genus *Anopheles* (Diptera: Culicidae). Malaria is caused by protozoan parasites (*Plasmodium* spp.) that enter the host’s bloodstream when female mosquitoes blood feed for egg development^2^. In the Republic of Korea (ROK), there are currently approximately 200–300 locally acquired malaria cases annually resulting from infections of *Plasmodium vivax*^3,4^. While there are imported cases of *Plasmodium falciparum*, autochthonous transmission has not been documented^5^.

Eight *Anopheles* species are found in the ROK, including six members belonging to the Hyrcanus Group (*Anopheles pullus* Yamada, *Anopheles belenrae* Rueda, *Anopheles sineroides* Yamada, *Anopheles lesteri* Baisas & Hu, *Anopheles kleini* Rueda, *Anopheles sinensis* sensu stricto Wiedemann; and one each belonging to the Barbirostris Group (*Anopheles koreicus* Yamada & Watanabe) and Lindesayi Group (*Anopheles lindesayi* Giles)^6,7^. Two species, *An. lesteri* and *An. kleini* are suspected to be the primary malaria vectors. However, there is much controversy concerning which species are the primary vectors of vivax malaria in the ROK^8–11^. Nevertheless, studies in the ROK have detected *P. vivax* in all species, except *An. koreicus* that is infrequently collected by mosquito traps^12–14^. Some members of the Barbirostris Group are major malaria vectors in some tropical regions^15^, such as Thailand^16^, suggesting the need to continue to monitor and control all *Anopheles* mosquitoes in the ROK. In the ROK, *An. sinensis* seems to have the widest distribution range among *Anopheles* mosquitoes^7,17^, and there are differences in seasonal occurrence of the species belonging to the Hyrcanus Group^14^. A survey of the larval habitat range conducted by Rueda et al.^18^ also found that *An. sinensis* was collected in the most diverse environments, while *An. koreicus* and *An. lindesayi* were only found primarily in forested areas^7,18,19^.

Effective malaria control is contingent on accurate surveillance of suspected malaria vector species. *Anopheles* mosquitoes are highly susceptible to the loss of primary morphological taxonomic key characteristics, e.g., wing scale patterns and leg spots, during their collection. In addition, *Anopheles* mosquitoes are morphologically extremely similar, making them difficult to identify to the species level^20^. This challenge is not limited to ROK *Anopheles* but also other *Anopheles* species in other continents, e.g., Africa^21^ and South America^22^. While molecular markers are currently being used for species identification of *Anopheles* spp., the process is time consuming and costly^23,24^. Therefore, more affordable and streamlined surveillance tools are necessary for timely monitoring *Anopheles* species.

The mitochondrial genome is a commonly used region for species identification. However, studies of the African malaria vector, *An*. *gambiae* complex, reported that mitochondrial markers are not sufficient to distinguish species within the complex^25^. Similarly, phylogenetic studies of mosquitoes belonging to the Hyrcanus Group have been performed using the cytochrome *c* oxidase subunit I (*COI*), cytochrome *c* oxidase subunit II (*COII*), and internal transcribed spacer 2 (ITS2) regions. However, the mitochondrial DNA (mtDNA) *COI* and *COII* regions are not useful for resolving phylogenetic relationships between *An. sinensis*, *An. kleini*, and *An. belenrae*^26,27^. The ITS2-based PCR method has been used for distinguishing species in the Maculipennis Group^28^, e.g., the *An. crucians* complex^29^ and others^30^. Although interspecific differences exist in the ITS2 region in the Hyrcanus Group, differences among *An. sinensis*, *An. kleini*, and *An. belenrae* are not substantial^24,27,31,32^.

The use of multiple nuclear DNA markers for species identification has an added benefit for investigating hybridization of closely related species^33,34^. Studies of African malaria vectors utilizing species-specific markers on three different chromosomes reveal a spectrum of degree in hybridization and adaptive introgression accelerating insecticide resistance selective sweep^33–35^. *Anopheles kleini* and *An. belenrae* were designated as new species in 2005^6^, and hybridization among *An. kleini* and *An. sinensis* are still known to occur^36,37^. Each genetic region is likely to diverge at different rates, and hence, comparing multiple genetic regions with varying functions will be useful for determining accurate evolutionary relationships^38^. Methods using multi-locus markers have been successfully used to accurately identify *Anopheles* species, e.g., members of the *An. gambiae* complex that is known to be capable of hybridization^33,39,40^.

Wing Geometric Morphometrics (WGM) is currently being used for the accurate species identification and ecological investigations of insects, and various tools have been developed that can be used by personnel unfamiliar with taxonomy^41,42^. In particular, the two-dimensional structure of the mosquito wing enables easy collection of landmarks (LMs), and the vein structure of the wing is species-specific making it cost effective and time-efficient compared with traditional methods of identification or using molecular markers^42^.

Phylogenetic signals are the tendency for closely related species to resemble each other more than evolutionarily distant species. Phylogenetic signals are now widely accepted and used in various ecological and evolutionary studies^43^. This analysis is being applied to test the congruence of morphometric data and molecular trees and is actively used in wing patterns of various insects^44,45^.

In this study, we investigated multiple approaches for *Anopheles* species identification, e.g., the potential use of wing shape and size for *Anopheles* species identification, in the ROK. The DNA barcoding region, *COI*, for comparison with WGM data was sequenced. Moreover, the partial sequence of tyrosine hydroxylase (*TH*) was obtained. This is a nuclear gene associated with the wing melanin pattern in *Drosophila* (Diptera: Drosophilidae)^46^, which also has important physiological functions, e.g., as cuticle tanning and innate immunity in *Anopheles* mosquitoes^47^. A molecular phylogenetic study of all *Anopheles* spp. in the ROK using the *TH* and *COI* regions in this study together with ITS2 obtained from GenBank was conducted and compared with the results of WGM analysis.

## Methods

### Sample collection and identification

**Figure 1:**
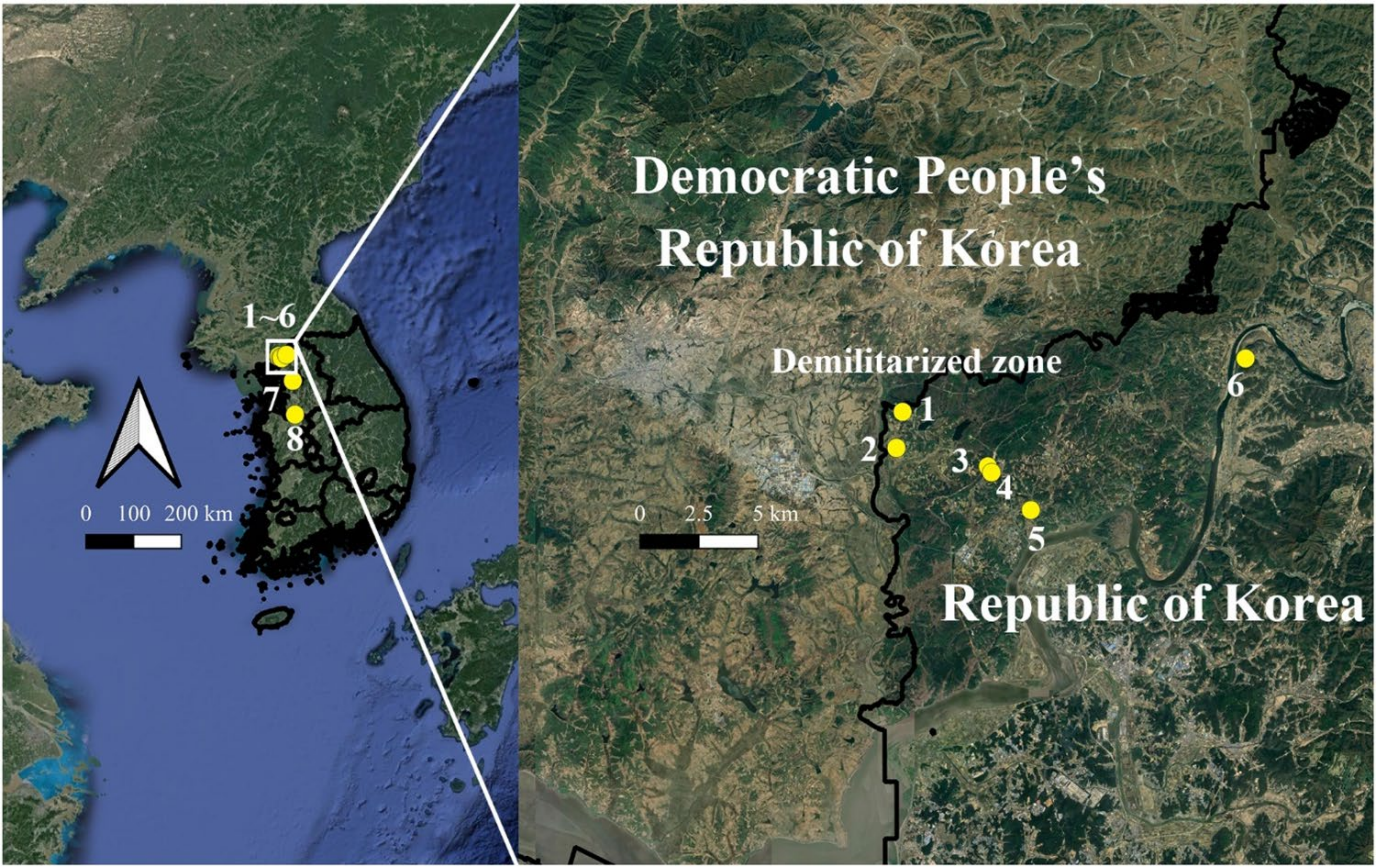
Collection sites of *Anopheles* mosquitoes in the ROK. 1. Neutral Nations Supervisory Commission camp (< 10 m from the DMZ), 2. Daeseong-dong (village inside the DMZ), 3. South gate entrance to the DMZ, 4. Camp Bonifas, 5. Warrior Base training area, 6. Dagmar North training area, 7. Yongsan USAG, 8. Humphreys USAG. This map was generated using Google earth Pro v.9.1.9 (https://earth.google.com) and QGIS 3.26.3 (https://www.qgis.org/ko/site).

Eight species of *Anopheles* mosquitoes were collected at six sites [(1) Neutral Nations Supervisory Commission camp (NNSC) (37° 57ʹ17.19″ N, 126° 40ʹ47.91″ E); (2) Daeseong-dong (37° 56ʹ28.31″ N, 126° 40ʹ37.38″ E); (3) South gate entrance to the DMZ (37° 56ʹ03.53″ N, 126° 43ʹ15.46″ E); (4) Camp Bonifas (37° 55ʹ55.25″ N, 126° 43ʹ21.73″ E), (5) Warrior Base training area (37° 55ʹ03.96″ N, 126° 44ʹ29.74″ E); and (6) Dagmar North training area (37° 58ʹ29.85″ N, 126° 50ʹ40.88″ E)] from April to October 2021 in/near the demilitarized zone (DMZ), a malaria high-risk area in the ROK, to reduce seasonal variation among individuals for WGM analysis. Additional collections were also conducted at Yongsan US Army Garrison (USAG) (Seoul) (37° 31ʹ56.2″ N, 126° 58ʹ53.4″ E) and Humphreys USAG (Pyeongtaek) (36° 57ʹ19.9″ N, 127° 01ʹ41.4″ E) (Fig. 1).

Mosquitoes were collected using Mosquito Magnets (Woodstream Corp., Lititz, PA, USA), returned to the central laboratory at Humphreys USAG where they were identified to species (*An. lindesayi*, *An. koreicus*, and *An. sineroides*) or genera, and then stored at − 70 °C. Mosquitoes were then transported on dry ice to Kyungpook National University, Daegu where they were stored at − 70 °C until used, molecular identification was performed using the hind leg^24^ (Table S1).

### Geometric morphometrics analysis

**Figure 2:**
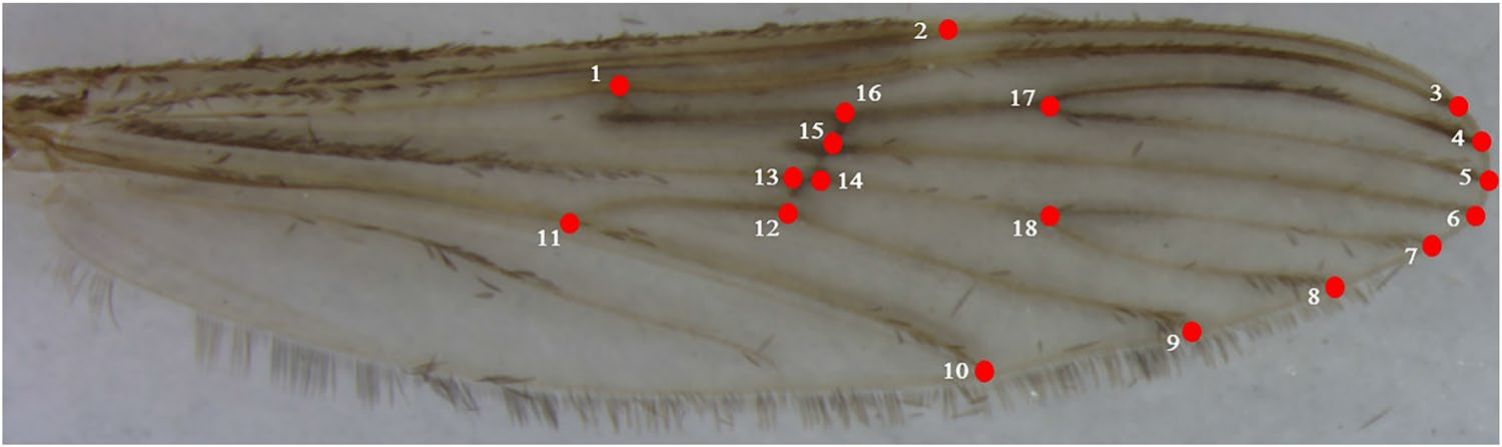
The 18 landmarks on the wing of *Anopheles* species.

WGM analysis was conducted using the right wings of eight *Anopheles* species that were identified using molecular methods. The right wing was removed scales removed using a paintbrush, then placed on a slide and then covered with a cover slip using Canada balsam (Duksan, Seoul, ROK). The wings of each *Anopheles* species were then photographed under magnification (× 20) using an Olympus SZ61 Stereo Microscope (Olympus Corp., Tokyo, Japan). For WGM analysis, 18 landmarks (LMs) were selected using TPSdig2 v.2.31^48^ (Fig. 2), and the LM data analyzed using R v.4.2.1^49^.

The thin plate spline (TPS) file with LM data was first analyzed using the geomorph v.4.0.5 package^50^. To generate Procrustes coordinates, the “gpagen” function was used to transform differences in the position and orientation of each data point, and the coordinates were superimposed. In this process, the centroid size (CS), defined as the square root of the sum of squared distances of all the LMs of an object from their centroid, was calculated as a proxy for the wing size of each species^51^. The allometric effect, which indicates the relationship between the CS and wing shape, was calculated using the “procD.lm” function with 1000 permutations. For statistical comparisons of the mean values of CS for each measured species, an analysis of variance (ANOVA) was performed, and multiple pairwise comparisons applied for the eight *Anopheles* species using the “LSD.test” function of the agricolae v.1.3-6 package, followed by t-tests (Bonferroni-adjusted p values)^52^. To determine the accuracy of the grouping of the eight *Anopheles* species based on wing shape, a linear discriminant analysis (LDA) using the “lda” function in the MASS v.7.3-60 package was performed^53^. Furthermore, the convex hull algorithm was applied, which is a method for including all data for each species in the scatterplot resulting from LDA.

Canonical variate analysis (CVA) was conducted using the “CVA” function in the Morpho v.2.11 package, and the Mahalanobis distance calculated using a statistical measure of morphological differences in each group^54^. To verify the accuracy of the reclassification based on wing shape, a pairwise cross-validation test (leave-one-cut method) based on canonical variate analysis was performed using the “CVA” and “typprobClass” function of the Morpho v.2.11 package^54^. Based on the Mahalanobis distance, a hierarchical clustering dendrogram was constructed using the “pvclust” function in the pvclust v.2.2-0 package^55^. To check whether the set number of LMs was oversampled, the “lasec” function of the LaMBDA v.0.1.1 package was used^56^. The morphological differences in the wings of *Anopheles* mosquitoes were determined by first evaluating the average wing shape of the eight species and then evaluating the average shape of each species, followed by visualization using the “plotRefToTarget” (method = points) function of the geomorph v.4.0.5package^50^. The results of the analysis using WGM were visualized using the ggplot2 v.3.4.2 package^57^.

### DNA sequencing

Genomic DNA was extracted from the entire body, except the right wing and hind legs, of the eight *Anopheles* species for *COI* and *TH*. DNA extraction was performed using the Clear-S™ Quick DNA Extraction Kit (InVirusTech, Gwangju, ROK) according to the manufacturer’s protocol. Universal primer pairs (LCO1490: 5′-GGT CAA CAA ATC ATA AAG ATA TTG G-3′/HCO2198: 5′-TAA ACT TCA GGG TGA CCA AAA AAT CA-3′) were used to amplify the *COI* region^58^. The reaction mixture for PCR amplification (total: 25 μL) consisted of 1× PCR buffer, 0.2 mM dNTPs, 1.5 mM MgCl_2_, 0.4 μM of each primer, 0.5 units *Taq* DNA polymerase (TaKaRa, Shiga, Japan), and 1 μL extracted genomic DNA. The PCR cycling conditions were as follows: initial denaturation at 94 °C for 5 min followed by 30 cycles at 94 °C for 30 s, 56 °C for 30 s, 72 °C for 1 min, and then a final extension at 72 °C for 5 min.

Universal primers for the *TH* gene region were constructed using the sequences of *An. sinensis* (Genbank accession numbers: KU886220, AXCK02010477) and *An. stephensi* (Genbank accession number: CP032302) registered in GenBank. Primers were designed to enable the combined amplification of the exon and intron regions (Kan_th_F: 5ʹ-GGT TCA ACA GAC AGT CGA GG-3′/Kan_th_R: 5ʹ-GTT GAC GTT GTT CTC CGA CA-3′; Kan_th_F2: 5ʹ-CTG CCC CAG AAG CCA GAG-3′/Kan_th_R2: 5ʹ-CTG CAA CAC AAC CTC CTC CT-3′). The reaction mixture for PCR amplification (total: 25 μL) consisted of 1 × PCR buffer, 0.2 mM dNTPs, 1.5 mM MgCl_2_, 0.4 μM of each primer, 0.5 units *Taq* hotstart DNA polymerase (TaKaRa, Shiga, Japan), and 1 μL extracted genomic DNA. The PCR cycling conditions were as follows: initial denaturation at 94 °C for 5 min followed by 35 cycles at 94 °C for 30 s, 58 ~ 60 °C for 40 s, 72 °C for 1 min, and then a final extension at 72 °C for 10 min.

To confirm whether the PCR amplification was successful, the amplification products were identified by electrophoresis on a 1.5% agarose gel, and the successfully amplified samples were subjected to Sanger dideoxy sequencing in both directions by Macrogen (Daejeon, ROK).

The sequences for each specimen were aligned and edited using BioEdit^59^ and then compared and confirmed using NCBI’s Basic Local Alignment Search Tool. The sequences for the *COI* and *TH* genes of the eight *Anopheles* species obtained in this study were deposited in the GenBank (GenBank accession numbers (*COI*): OR150355-OR150394; GenBank accession numbers (*TH*): OR187248–OR187297).

### Phylogenetic analysis

Three regions (*COI*, *TH*, and ITS2) were analyzed for the molecular phylogenetic analysis (*COI*: 615 bp; *TH*: 656 bp; ITS2: 538 bp). The registered sequences in GenBank were used for the analysis of the ITS2 region (Table S2). Sequence alignments for each region of the eight *Anopheles* species were made using the L-INS-i method in MAFFT v.7^60^. Next, a maximum likelihood (ML) tree was generated for the phylogenetic analysis of each sequence. The R’s phangorn v.2.11.1 and ape v. 5.7-1 packages were used for nucleotide substitution model selection and bootstrap analysis for each locus^61,62^. After checking with the “modelTest” function, *COI*: GTR + G (4) + I; ITS2, *TH*: GTR + G (4) were found to be the best fit models. The “bootstrap.pml” function with 1000 replications were applied to check the support for a node in the tree. Consensus sequences for each species were generated using the “ConsensusSequence” function in the DECIPHER v.2.24.0 package^63^. The relative nucleotide diversity (π) for the exon and intron of the *TH* gene using the “nuc.div” function of the pegas v.1.2 package was also calculated^64^. The results obtained from this phylogenetic analysis were visualized using the ggtree v.3.4.4 and ggmsa v.1.3.4 packages^65,66^.

In addition, evolutionary phylogenetic inferences about the CS and wing shape of the eight *Anopheles* species analyzed by WGM were drawn using the geomorph v.4.0.5 and phytools v.1.5-1 packages^50,67^, for which a phylogenetic tree based on the *TH* gene region of this study was used. According to the Brownian model of evolutionary change, species diverge over time, and the similarity or dissimilarity of variation between species can be deduced by the phylogenetic signal^68^. To identify these phylogenetic signals, the “physignal” function in geomorph v.4.0.5 package with 10,000 permutations was used to determine whether a phylogenetic signal existed in the Procrustes shape variables^50^. To visualize the quantitative morphometric data in a phylogenetic context, the phylogenetic signals for CS and wing shape were projected onto a phylogenetic tree using the “contMap” function in phytools v.1.5.-1 package and “gm.prcomp” function in geomorph v.4.0.5 package, respectively^50,67^.

## Results

**Figure 3:**
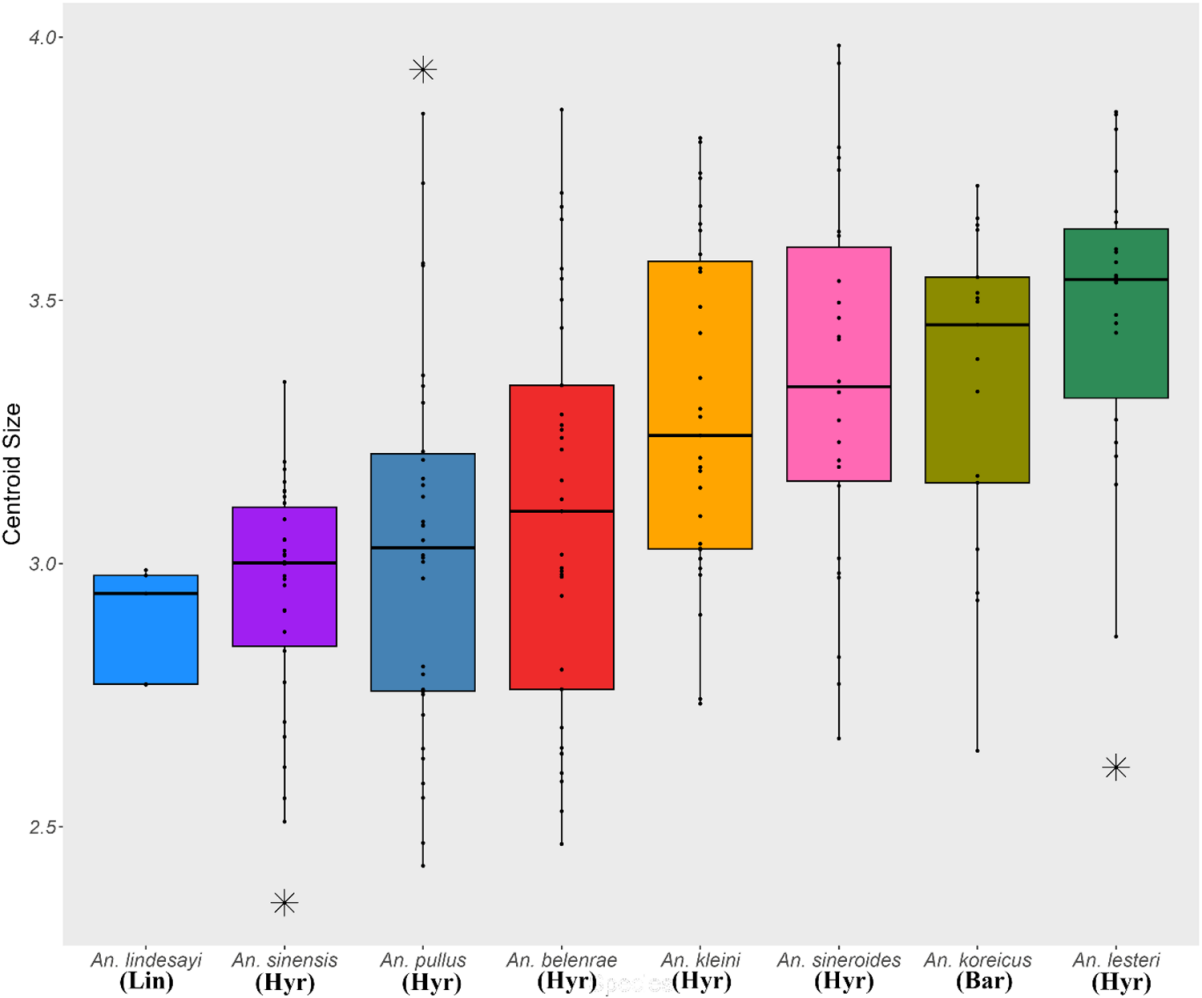
Boxplot showing the results of centroid size comparisons for each *Anopheles* species present in the ROK. Lin, Hyr, and Bar refer to the Lindesayi Group, Hyrcanus Group, and Barbirostris Group, respectively. The box represents the first and third quartiles, and the black line in the middle represents the median. Black stars indicate outliers.

Mosquitoes were collected over several months for WGM analysis to minimize seasonal variation in their wings. First, the wing shape and CS exhibited small but significant relationships (R^2 ^= 1.8%, *P* = 0.0006). However, because wing size can also be useful for species identification, the data obtained were used in further analyses. Comparisons of wing size as a proxy for CS demonstrated differences in mean CS between groups of *Anopheles* mosquitoes of each species (ANOVA, F = 8.234, *P* < 0.001). Among the eight *Anopheles* species, *An. lesteri* had the largest CS, whereas *An. lindesayi* had the smallest CS (Fig. 3). However, when analyzed by pairwise comparisons, no significant size differences were observed in CS for most species (Table S3).

**Figure 4:**
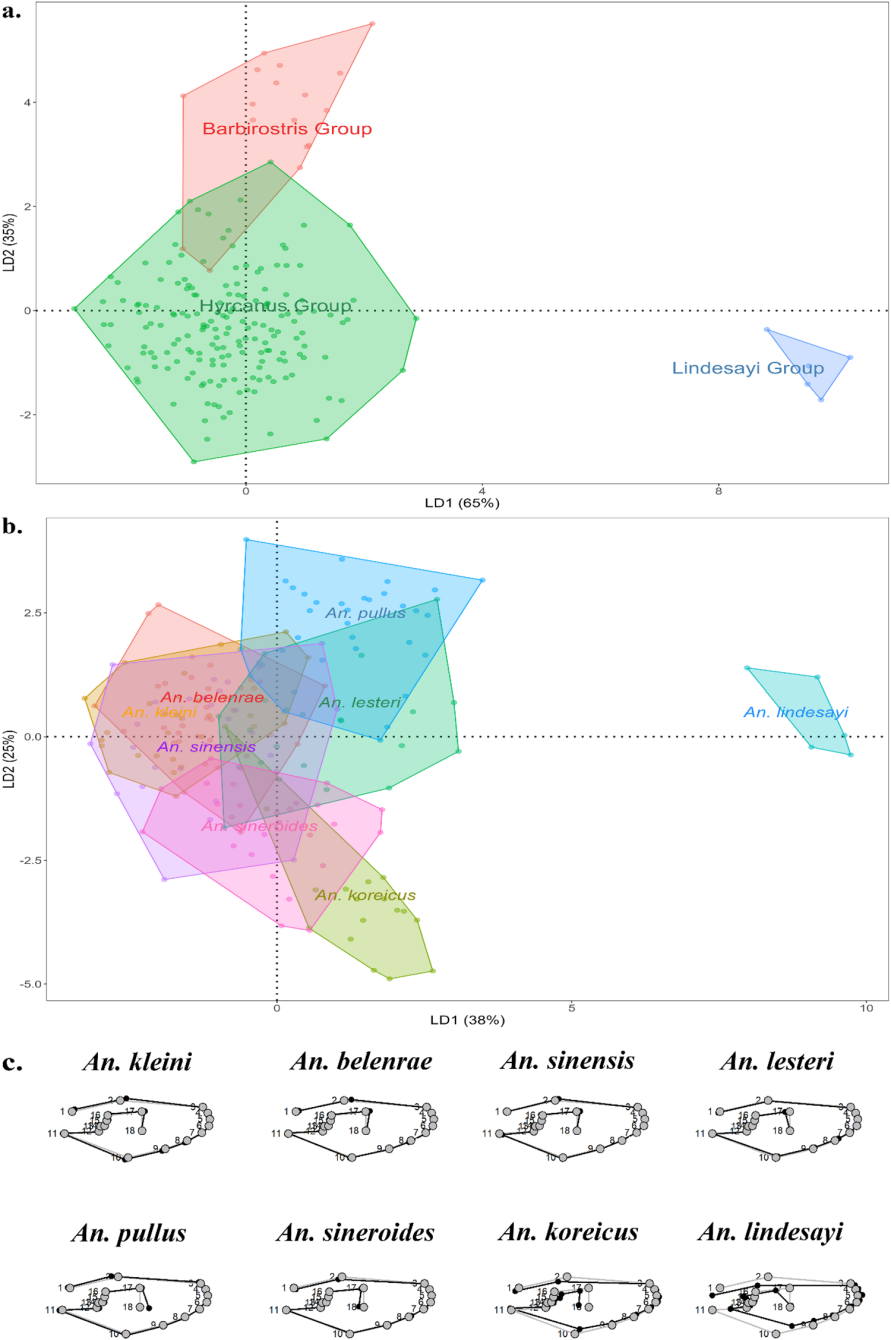
(**a**) Scatterplot of Linear Discriminant Analysis (LDA) results based on wing shape variation from the three groups (LD1: 65%, LD2: 35%). (**b**) Scatterplot of LDA results based on wing shape variation from the eight *Anopheles* species (LD1: 38%, LD2: 25%). (**c**) Comparison of mean shapes for *Anopheles* mosquitoes. The gray line is the average shape for all eight species, and the black line is the average shape for each species (magnified by 3×).

LDA analysis of wing shape data based on each group confirmed that the Hyrcanus and Barbirostris Groups, which are known to be more closely related than the Lindesayi Group, were in close proximity based on the scatterplot (Fig. 4a). In the scatterplot representing the results of the analysis at the species level, a strong overlap between *An. sinensis*, *An. belenrae*, and *An. kleini* was observed (Fig. 4b). When the average wing shape of the eight species was evaluated and compared (Fig. 4c), six mosquito species belonging to the Hyrcanus Group showed variations primarily between LMs 17 and 18. However, the differences among members of the Hyrcanus Group were not large compared with the average morphological differences of all eight species, whereas *An. koreicus* (Barbirostris Group) and *An. lindesayi* (Lindesayi Group) exhibited large differences in LMs 10 and 11, LMs 15 and 16, and LMs 17 and 18. The pairwise cross-validation test (leave-one-cut method) was performed to evaluate the reclassification accuracy, and the results showed a high level of accuracy at the group level [Overall accuracy: 94.4% (Hyrcanus Group: 94.9%; Barbirostris Group: 88.2%; Lindesayi Group: 100%)]. In contrast to the comparison at the group level, the pairwise cross-validation test at the species level showed a lower reclassification rate (Overall accuracy: 75.8%). Results of the analysis at the species level, *An. pullus* could be recognized with 88.2% accuracy, and *An. sineroides* could be identified with 80.9% accuracy. However, *An. belenrae* and *An. lesteri*, which belong to the Hyrcanus Group, showed the lowest classification accuracy with 57.6% and 68.1% accuracy, respectively (Table 1). This was observed especially for *An. belenrae* where many specimens were misidentified as *An. sinensis* or *An. kleini*. The LMs for potential oversampling were checked and the 18 LMs established for WGM analysis were not oversampled (fit = 0.90: 14 LMs; fit = 0.95: 16 LMs; fit = 0.99: 17 LMs) (Fig. S1).

**Table 1:** Results of pairwise cross-validation test (leave-one-cut method) for eight *Anopheles* species based on wing shape (Overall accuracy: 75.8%).

Species	*An. kleini* (%)	*An. belenrae* (%)	*An. sinensis* (%)	*An. lesteri* (%)	*An. pullus* (%)	*An. sineroides* (%)	*An. koreicus* (%)	*An. lindesayi* (%)
*An. kleini*	77.4	12.9	6.5	0.0	0.0	3.2	0.0	0.0
*An. belenrae*	21.2	57.6	15.2	3.0	0.0	3.0	0.0	0.0
*An. sinensis*	13.3	6.7	70.0	0.0	3.3	6.7	0.0	0.0
*An. lesteri*	4.5	9.2	4.5	68.1	9.2	4.5	0.0	0.0
*An. pullus*	0.0	0.0	5.9	5.9	88.2	0.0	0.0	0.0
*An. sineroides*	7.7	3.8	3.8	0.0	0.0	80.9	3.8	0.0
*An. koreicus*	5.9	0.0	0.0	0.0	0.0	5.9	88.2	0.0
*An. lindesayi*	0.0	0.0	0.0	0.0	0.0	0.0	0.0	100

**Figure 5:**
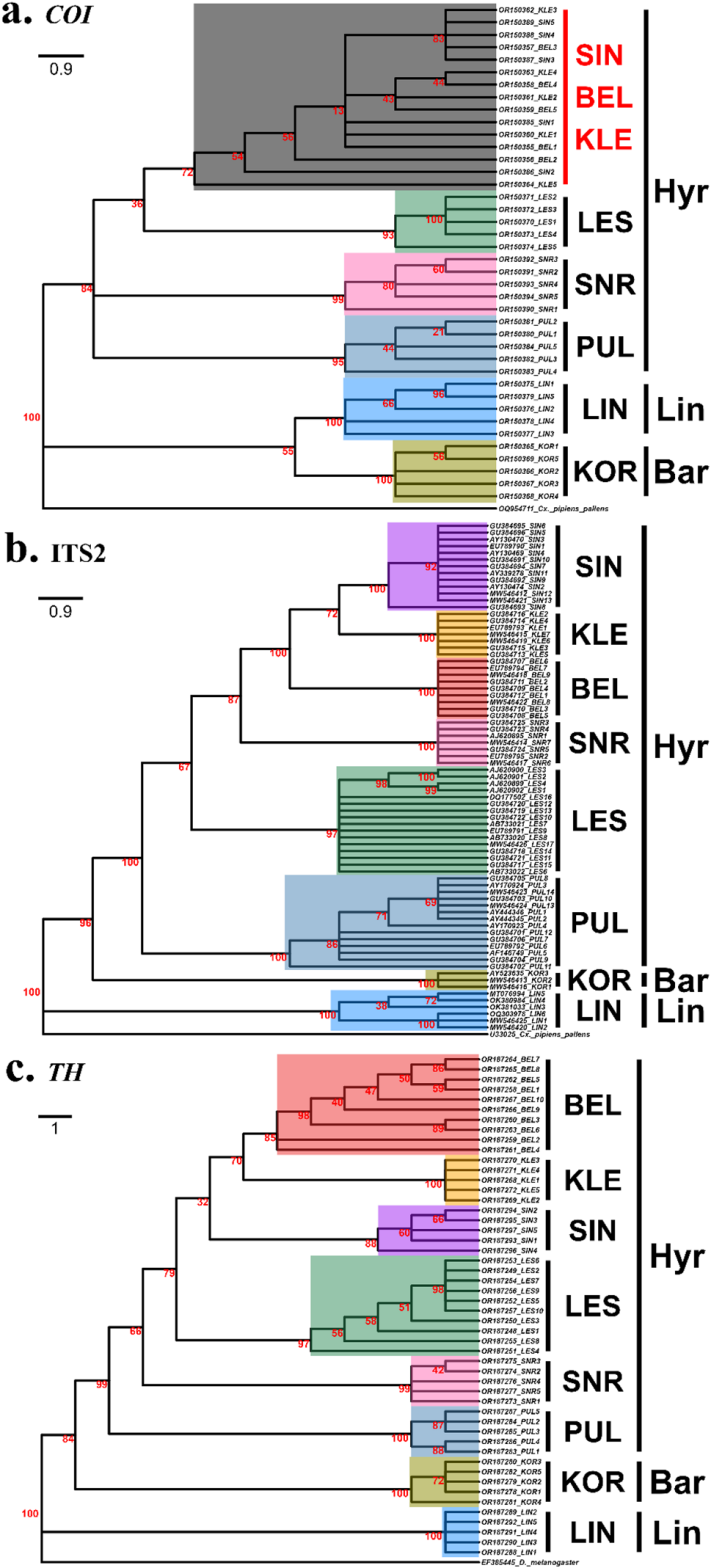
Simplified maximum likelihood (ML) phylogenetic trees constructed in this study using *COI*, ITS2, and *TH* gene fragments (SIN = *An. sinensis*, BEL = *An. belenrae*, KLE = *An. kleini*, LES = *An. lesteri*, SNR = *An. sineroides*, PUL = *An. pullus*, KOR = *An. koreicus*, LIN = *An. lindesayi*). (**a**) ML tree based on *COI* regions [GTR + G (4) + I)]. (**b**) ML tree based on ITS2 regions [GTR + G (4)]. (**c**) ML tree based on *TH* gene [GTR + G (4)]. Bootstrap was performed with 1000 replications, and the value is represented on the node in red. Unresolved clustering is highlighted in red. Hyr, Lin and Bar refer to the Lindesayi Group, Hyrcanus Group, and Barbirostris Group, respectively.

**Figure 6:**
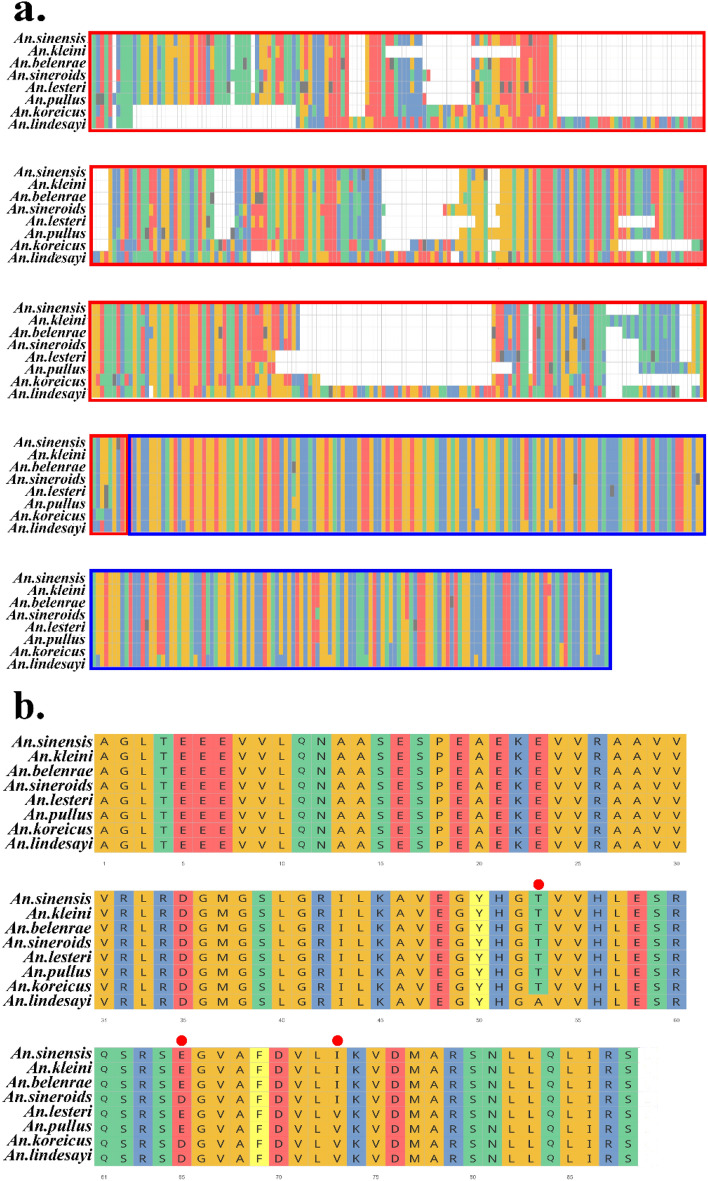
(**a**) Multiple nucleotide sequence alignments of the *TH* gene fragment (red: A, blue: C, green: T, yellow: G, black: ambiguous nucleotide). Red boxes represent intron regions, and blue boxes represent exon regions. (**b**) Multiple amino acid sequence alignments of *TH* gene. Red circles indicate areas of variation.

An ML tree was generated for molecular phylogenetic inference using the *COI* regions of the eight *Anopheles* species in the ROK obtained during this study (Fig. 5a). Consistent with previous results^26^, analyses using *COI* reaffirmed that the relationships of *An. sinensis*, *An. belenrae*, and *An. kleini* in the Hyrcanus Group were unclear to resolve. However, using the ITS2 region, all eight species were clearly separated, including *An. sinensis*, *An. belenrae*, and *An. kleini*, which were not accurately identified in the analysis using the *COI* region (Fig. 5b). For the tree based on the *TH* region, the topology did not match the tree constructed based on the ITS2 region, but all eight species were separated (Fig. 5c). In particular, interspecific differences were evident primarily in the intron region of the *TH* gene (Fig. 6a), whereas no substantial sequence differences were observed in the exon region (Fig. 6b). The nucleotide diversity for the exon and intron *TH* gene was evaluated, respectively, and found that the value of π in the exon was 0.0204 (n = 50), whereas that in the intron was 0.1699 (n = 50), indicating much larger nucleotide diversity in the intron.

**Figure 7:**
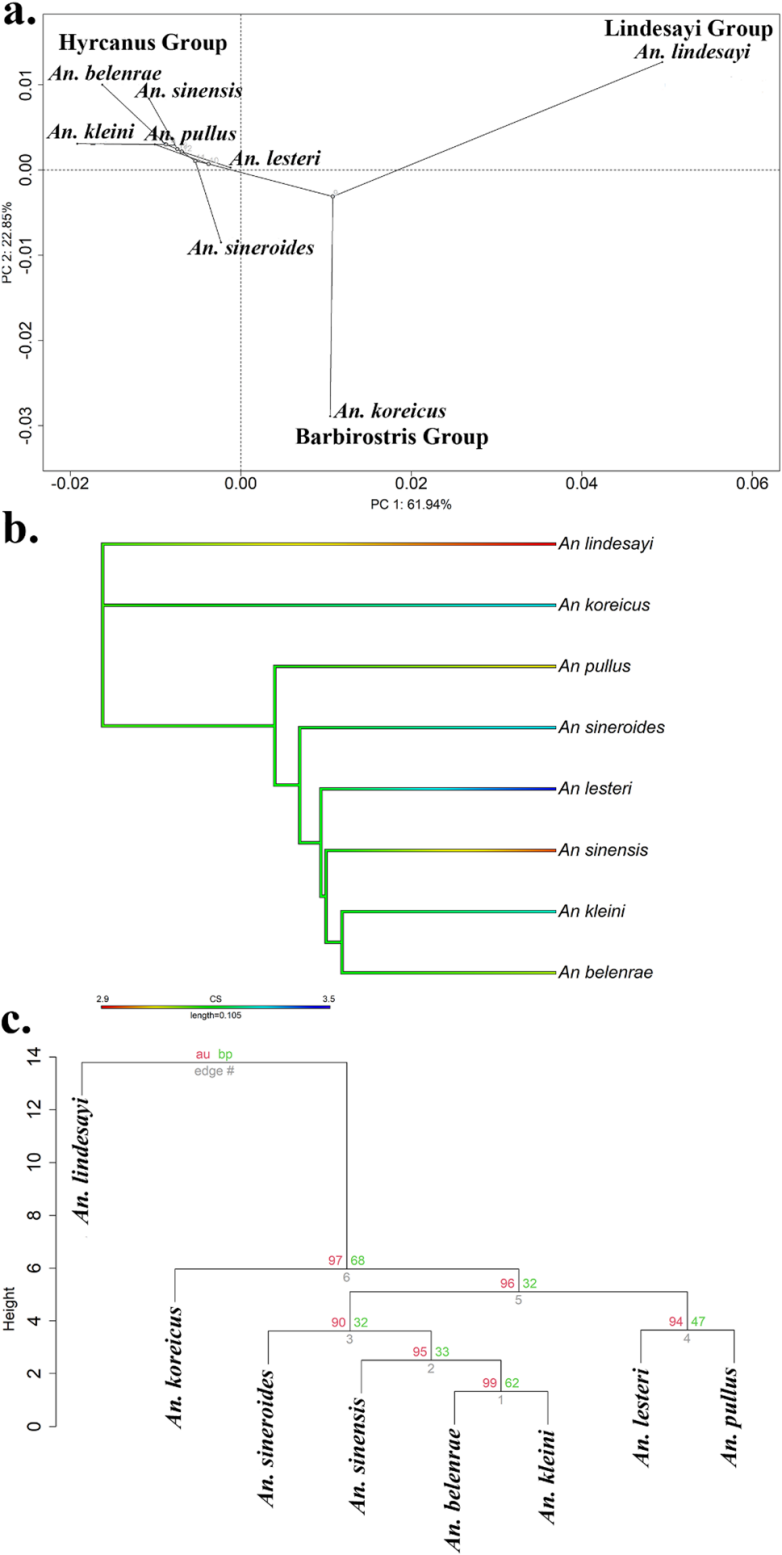
(**a**) Phylomorphospace based on wing shapes of the eight *Anopheles* species present in the ROK (PC1: 61.94%, PC2: 22.85%). (**b**) CS evolution of *Anopheles* mosquitoes in the ROK. Wing shape and CS were projected onto the *TH* gene tree constructed in this study. (**c**) Dendrogram based on Mahalanobis distance between the eight *Anopheles* species in the ROK. Values at branches are approximately unbiased (au) *p*-value (red), bootstrap probabilities (bp) (green) with 1000 bootstrap replicates.

After analysis of the phylogenetic signal for wing shape, we found a strong phylogenetic signal in shape (K = 1.4, *P* = 0.01). A strong phylogenetic signal indicates that the wing shape evolved from a shared evolutionary history, rather than environmental factors^43,68,69^. As illustrated by the phylomorphospace formed based on wing shape (PC1: 61.94%, PC2: 22.85%), the six species belonging to the Hyrcanus Group were closely positioned to each other, whereas *An. koreicus* and *An. lindesayi*, which belong to different groups, are far diverged from each other in the phylomorphospace (Fig. 7a). In contrast to wing shape, no significant phylogenetic signal was observed in the CS (K = 0.41, *P* = 0.31) (Fig. 7b). In the hierarchical clustering dendrogram, *An. lesteri* and *An. pullus* clustered together, and the other four species in the Hyrcanus Group clustered together (Table S4, Fig. 7c). For *An. koreicus* and *An. lindesayi*, which do not belong to the Hyrcanus Group, a separate branch was formed.

## Discussion

*Anopheles* specimens collected by Mosquito Magnet traps during the mosquito season from April–October were used for WGM analysis. Our results showed that CS was not a useful phenotypical trait for the identification of *Anopheles* species in the ROK. The CS did not demonstrate any statistically significant differences for most of the *Anopheles* species present in the ROK. However, wing size is highly influenced by environmental factors, e.g., temperature and humidity. Therefore, caution is necessary when interpreting CS results using WGM analysis^70,71^.

LDA analysis based on wing shape confirmed that each *Anopheles* mosquito demonstrates species-specific venation at the group level (Hyrcanus Group vs. Barbirostris Group vs. Lindesayi Group). Each mosquito species showed a considerable amount of variation, primarily in LM 15–18. In this study, LM15 is located in radius 4 plus 5, LM16, 17 are located in radius 2 plus 3, and LM18 is located in media 1 plus 2^72^. A study comparing the wings of *An. dirus* and *An. baimaii*^73^ and the *An. barbirostris* complex^74^ found differences primarily in radius 2 plus 3 and media 1 plus 2, similar to our results. Based on the results of this study and previous studies^73,74^, it appears that the variation in LM is mainly found in radius 2 plus 3 and media 1 plus 2, which will need to be evaluated among more *Anopheles* species in the future. Wing venation is an important marker for species identification and has been shown to exert potentially important aerodynamic effects in insect wings^75–77^. In the case of mosquitoes, each species exhibits different ecological characteristics (endophilic or exophilic; stenogamous or eurygamous) with large differences in host preference (e.g., mammal- or avian-feeding). Different ecological characteristics of each species may have exerted selective pressure on wing venation, which was reflected in the eight *Anopheles* spp. that exhibited differences in venation. However, it is difficult to determine whether the variation in wing shape at the group and species level is due to ecological features since there is a lack of ecological research data on *Anopheles* mosquitoes present in the ROK. Future studies that link morphological data, such as WGM, to ecological features will provide more information. At the present time, only one species each of the Lindesayi and Baribirostris Groups of mosquitoes is known to occur in the ROK^7,12–14^. Therefore, further studies should include more mosquitoes from the Lindesayi and Barbirostris Groups.

In this study, data were obtained for the *TH* and *COI* gene fragments and a molecular phylogeny was inferred using the three regions (*TH*, *COI*, and ITS2). Based on *COI*, the mosquito species in each of the three groups were monophyletic, but it was difficult to determine the phylogenetic relationships for *An. sinensis*, *An. kleini*, and *An. belenrae* in the Hyrcanus Group. In the case of *An. sinensis* and *An. kleini*, hybridization occurred under the experimental conditions, and similarly, *An. sinensis* and *An. kleini*, as well as *An. belenrae*, might have experienced ancient hybridization^36,37^. Unlike the analysis based on the mtDNA *COI* region, the analysis based on nuclear DNA ITS2 and *TH* gene fragments resolved all three species (*An. sinensis*, *An. kleini*, and *An. belenrae*). The *COI* region is actively used as a barcoding region for species identification^78^, and it is generally believed that the mitochondrial genome diverges at a faster rate than the nuclear genome^79^. However, the analysis based on *TH* + ITS2 (nuclear DNA) and *COI* (mtDNA) in this study and previous studies for members of the Hyrcanus Group suggests a contradiction in some species belonging to the Hyrcanus Group. Some reports suggest that these results are due to male-biased dispersal^26,27,32,80^, but whether the lower diversity of the mitochondrial genome than that of the nuclear genome is due to male-biased dispersal needs verification using various genetic tools and field observations^81^. Moreover, *Anopheles* mosquitoes within the Hyrcanus Group present in the ROK had an inconsistent topology between the *TH* and ITS2 trees, which is likely due to different rates of divergence for different genes that are dependent upon selection pressures^38,82^. For closely related species, such as *An. sinensis*, *An. kleini*, and *An. belenrae*, genome-wide data will be required to establish true phylogenetic tree.

For the *TH* gene fragment used for phylogenetic analysis, there was much greater nucleotide diversity in the intron regions than in exon regions. Currently, various nuclear genes, except *COI* and ITS2, are considered genetic markers to distinguish *Anopheles* species^83,84^. However, the *TH* gene fragment may also be used as a molecular marker for members of the Hyrcanus Group in the ROK. Conserved regions of exon are ideal for PCR primers, and the high divergence of intron sites may be useful for determining the phylogenetic relationships.

The hierarchical clustering dendrogram constructed using Mahalanobis distance based on wing shape, demonstrated similar results compared to molecular phylogeny. This was especially observed in the clustering of closely related species, e.g., *An. sinensis*, *An. kleini*, and *An. belenrae*, in the dendrogram and similar to the tree created using the ITS2 and *TH* gene fragments. Phylogenetic analysis using morphometric data is generally recognized to have some limitations compared to phylogenetic trees using molecular data, as studies have demonstrated that there is incongruence between molecular and morphometric analyses caused by homoplasy^85^. The analysis of wing shape used in our study demonstrated similar results as the molecular analysis for some species. Whether this pattern extends to other morphological features, as well as wings, will need to be compared with additional morphometrics data. No significant phylogenetic signals were identified in the CS of the eight *Anopheles* spp. used in this study. However, there was a strong phylogenetic signal identified using wing shape, which was confirmed by clustering of each group using phylomorphospace. These data indicate that closely related species have similar wing shapes compared with their distant relatives^68,86^. However, the presence of a strong phylogenetic signals in wing shapes does not mean that morphological comparisons using wing shapes can be used to make accurate phylogenetic inferences^85,87,88^. Consequently, further analysis of various anatomical features is necessary to validate the congruence between morphometric data and molecular phylogenetic analysis.

## Conclusion

Currently, there are more than 200 annual cases of vivax malaria in the ROK, mostly near the DMZ in Gyeonggi and Gangwon provinces adjacent to the Democratic People’s Republic of Korea (DPRK)^89,90^. *Anopheles kleini* is more frequently collected in human environments^89^. Successful disease vector mosquito control requires accurate species identification. For this purpose, a comparative analysis of all *Anopheles* mosquito species in the ROK through quantitative morphological and molecular phylogenetic analysis was conducted. This study confirmed that WGM can be used to identify with high accuracy at the group level but not sufficient for species identification. These results may assist a wide variety of personnel who are unfamiliar with traditional mosquito taxonomy or have difficulty with molecular approaches. The study also identified the *TH* gene as a candidate for a new genetic marker in addition to previously used *COI* and ITS2 molecular markers. *TH* gene genealogy was different from ITS2 genealogy and may be useful for phylogenetic studies for various *Anopheles* mosquito species where it is difficult to analyze the *COI* and ITS2 regions alone. The inclusion of more *Anopheles* species, not included in this study, would allow for more rigorous evolutionary comparisons and inferences between morphometric and molecular data.

### Supplementary Information

Supplementary Information.

## Data Availability

All sequencing data used in this research were deposited in the GenBank https://www.ncbi.nlm.nih.gov/under the accession numbers (*COI*: OR150355-OR150394; *TH*: OR187248-OR187297).
