# Supplementary material for: Analysis of geometric morphometrics and molecular phylogeny for Anopheles species in the Republic of Korea
Source: Sci Rep. 2023 Dec 12;13:22009. doi: 10.1038/s41598-023-49536-w (PMC10716165; doi:10.1038/s41598-023-49536-w)
Supplement: Supplementary file 1 — Supplementary Information. [file 41598_2023_49536_MOESM1_ESM.docx]

**Analysis of Geometric Morphometrics and Molecular Phylogeny for *Anopheles* Species in the Republic of Korea**

Jiseung Jeon^1,2,3^, Heung Chul Kim^4^, Terry A. Klein^5,6^ and Kwang Shik Choi^1,2,3,7^**^*^**

^1^ **School of Life Sciences, BK21 FOUR KNU Creative BioResearch Group,** **Kyungpook National University, Daegu 41566, Republic of Korea**

^2^ School of Life Sciences, College of Natural Sciences, Kyungpook National University, Daegu 41566, **Republic of Korea**

^3^ Research Institute for Dok-do and Ulleung-do Island, Kyungpook National University, Daegu 41566, **Republic of Korea**

**^4^** U Inc., Daesakwan-ro 34-gil, Yongsan-gu, Seoul, 04409, Republic of Korea

^5^ Force Health Protection and Preventive Medicine, Medical Department Activity-Korea/65th Medical Brigade, Unit 15281, APO AP 96281-5281, USA

^6^ PSC 450, Box 75R, APO AP 96206, USA

^7^ Research Institute for Phylogenomics and Evolution, Kyungpook National University, Daegu 41566, **Republic of Korea**

***Correspondence:** *ksc@knu.ac.kr*

School of Life Sciences, Kyungpook National University, Daegu 41566, **Republic of Korea**

Tel: +82-53-950-5351, Fax: +82-53-953-3066

**Supplementary materials**

**Fig. S1.** Landmark sampling evaluation curve using the “lasec” function (package LaMBDA). Gray line represents one iteration with a total of 1,000 iterations. Dark line is the average value (fit = 0.90: 14 LMs; fit = 0.95: 16 LMs; fit = 0.99: 17 LMs).

**Table S1.** *Anopheles* species collected from eight sites in the ROK between April and October in 2021 used in this study.

**Table S2**. ITS2 regions of the eight *Anopheles* species in the ROK that were used in this study.

**Table S3.** P-value representing the results of multiple pairwise comparisons for the CS for each of the eight *Anopheles* species in the ROK [calculations were validated using t-tests (Bonferroni-adjusted P value)]

**Table S4.** Mahalanobis distance based on the wing shapes of eight *Anopheles* mosquitoes in the ROK.


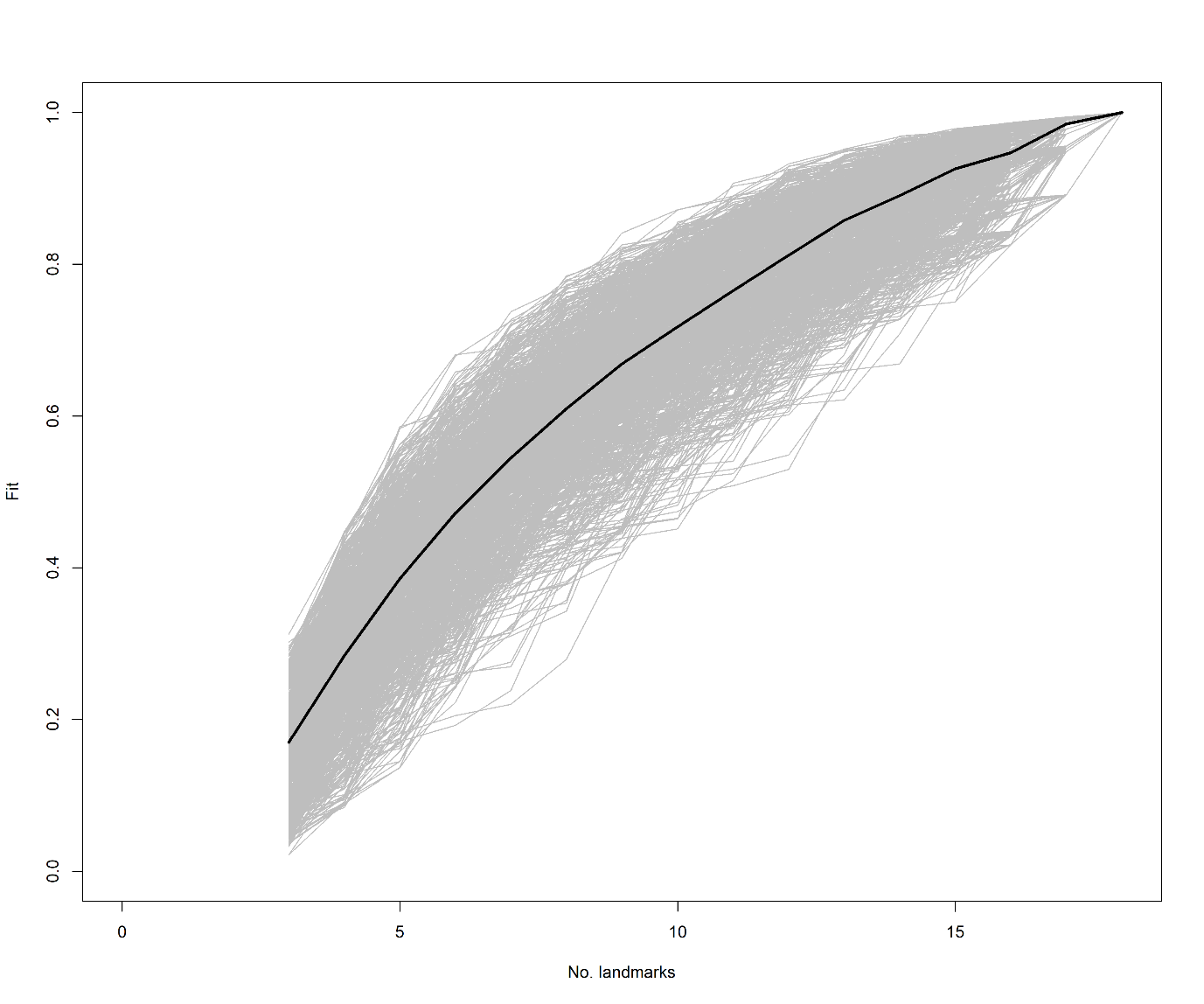


**Fig. S1.** Landmark sampling evaluation curve using the “lasec” function (package LaMBDA). Gray line represents one iteration with a total of 1,000 iterations. Dark line is the average value (fit = 0.90: 14 LMs; fit = 0.95: 16 LMs; fit = 0.99: 17 LMs).

**Table S1.** *Anopheles* species collected from eight sites in the ROK between April and October in 2021 used in this study.

| Species | Collection sites | | | | | | | | Total |
| --- | --- | --- | --- | --- | --- | --- | --- | --- | --- |
|  | NNSC | Daeseong-dong | South gate | Camp Bonifas | Warrior Base | Dagmar North | Yongsan | Humphreys |  |
| *An. pullus* | 2 | 3 | 8 | - | 2 | 10 | - | 9 | 34 |
| *An. belenrae* | 7 | 15 | 1 | - | 7 | 2 | - | 1 | 33 |
| *An. sineroides* | 1 | 1 | 3 | 2 | 3 | 16 | - | - | 26 |
| *An. lesteri* | 2 | 1 | 2 | - |  | 3 | - | 14 | 22 |
| *An. kleini* | 8 | 4 | 5 | 3 | 7 | 4 | - | - | 31 |
| *An. sinensis* | - | 7 | - | 2 | 7 | 1 | 4 | 9 | 30 |
| *An. koreicus* | 11 | 2 | - | 3 | 1 | - | - | - | 17 |
| *An. lindesayi* | 1 | - | 2 | - | - | 1 | 1 | - | 5 |

**Table S2.** ITS2 regions of the eight *Anopheles* species in the ROK that were used in this study.

| Species | Genbank accession number |
| --- | --- |
| ***An. pullus*** | AY444346  AY444345  AY170924  AY170923  AF146749  EU789792  GU384706  GU384705  GU384704  GU384703  GU384702  GU384701  MW546424  MW546423 |
| ***An. belenrae*** | GU384712  GU384711  GU384710  GU384709  GU384708  GU384707  EU789794  MW546422  MW546418 |
| ***An. sineroides*** | AJ620895  EU789795  GU384725  GU384723  GU384724  MW546417  MW546414 |
| ***An. lesteri*** | AJ620902  AJ620901  AJ620900  AJ620899  AB733022  AB733021  AB733020  EU789791  GU384722  GU384721  GU384720  GU384719  GU384718  GU384717  DQ177502  MW546426 |
| ***An. kleini*** | EU789793  GU384716  GU384715  GU384714  GU384713  MW546419  MW546415 |
| ***An. sinensis*** | EU789790  AY130474  AY130470  AY130469  GU384696  GU384695  GU384694  GU384693  GU384692  GU384691  AY339278  MW546412  MW546421 |
| ***An. koreicus*** | MW546416  MW546413  AY523635 |
| ***An. lindesayi*** | MW546425  MW546420  OK381033  OK380984  MT076994  OQ303978 |

**Table S3**. P-values representing the results of multiple pairwise comparisons for the CS of each of the eight *Anopheles* species in the ROK [calculations were validated using t-tests (Bonferroni-adjusted P value)].

| Species | *An. belenrae* | *An. sinensis* | *An. lesteri* | *An. pullus* | *An. sineroides* | *An. koreicus* | *An. lindesayi* |
| --- | --- | --- | --- | --- | --- | --- | --- |
| *An. kleini* | 0.508 | <0.001 | 1.000 | 0.089 | 1.000 | 1.000 | 0.342 |
| *An. belenrae* |  | 1.000 | 0.002 | 1.000 | 0.106 | 0.426 | 1.000 |
| *An. sinensis* |  |  | <0.001 | 1.000 | <0.001 | 0.003 | 1.000 |
| *An. lesteri* |  |  |  | <0.001 | 1.000 | 1.000 | 0.016 |
| *An. pullus* |  |  |  |  | 0.016 | 0.098 | 1.000 |
| *An. sineroides* |  |  |  |  |  | 1.000 | 0.136 |
| *An. koreicus* |  |  |  |  |  |  | 0.236 |

**Table S4.** Mahalanobis distance based on the wing shapes of eight species of *Anopheles* mosquitoes in the Republic of Korea.

| Species | *An. belenrae* | *An. sinensis* | *An. lesteri* | *An. pullus* | *An. sineroides* | *An. koreicus* | *An. lindesayi* |
| --- | --- | --- | --- | --- | --- | --- | --- |
| *An. kleini* | 1.9502 | 2.3069 | 3.8249 | 4.0102 | 3.3799 | 5.2733 | 11.4619 |
| *An. belenrae* |  | 2.4905 | 3.7603 | 4.2298 | 3.1931 | 5.5828 | 10.9381 |
| *An. sinensis* |  |  | 3.4857 | 4.2315 | 3.1505 | 5.2833 | 11.0506 |
| *An. lesteri* |  |  |  | 3.6607 | 3.6372 | 4.7930 | 10.1389 |
| *An. pullus* |  |  |  |  | 4.7270 | 5.6494 | 10.1461 |
| *An. sineroides* |  |  |  |  |  | 3.7979 | 10.5110 |
| *An. koreicus* |  |  |  |  |  |  | 10.6447 |
